# Association between Single Nucleotide Polymorphisms in Vitamin D Receptor Gene Polymorphisms and Permanent Tooth Caries Susceptibility to Permanent Tooth Caries in Chinese Adolescent

**DOI:** 10.1155/2017/4096316

**Published:** 2017-11-12

**Authors:** Miao Yu, Qian-Zhou Jiang, Zhe-Yi Sun, Yuan-Yuan Kong, Zhi Chen

**Affiliations:** ^1^The State Key Laboratory Breeding Base of Basic Science of Stomatology (Hubei-MOST) and Key Laboratory of Oral Biomedicine Ministry of Education, School and Hospital of Stomatology, Wuhan University, Wuhan, China; ^2^Key Laboratory of Oral Medicine, Guangzhou Institute of Oral Disease, Stomatology Hospital of Guangzhou Medical University, Guangzhou 510140, China

## Abstract

**Purpose:**

Dental caries is a multifactorial infectious disease. In this study, we investigated whether single nucleotide polymorphisms (SNPs) in vitamin D receptor (VDR) gene were associated with susceptibility to permanent tooth caries in Chinese adolescents.

**Method:**

A total of 200 dental caries patients and 200 healthy controls aged 12 years were genotyped for* VDR* gene polymorphisms using the PCR-restriction fragment length polymorphism (PCR-RFLP) assay. All of them were examined for their oral and dental status with the WHO criteria, and clinical information such as the Decayed Missing Filled Teeth Index (DMFT) was evaluated. Genomic DNA was extracted from the buccal epithelial cells. The four polymorphic SNPs (*Bsm *I,* Taq *I,* Apa* I, and* Fok *I) in* VDR* were assessed for both genotypic and phenotypic susceptibilities.

**Results:**

Among the four examined* VDR* gene polymorphisms, the increased frequency of the CT and CC genotype of the* Fok *I* VDR* gene polymorphism was associated with dental caries in 12-year-old adolescent, compared with the controls (*X*^2^ = 17.813, *p* ≤ 0.001). Moreover,* Fok *I polymorphic allele C frequency was significantly increased in the dental caries cases, compared to the controls (*X*^2^ = 14.144, *p* ≤ 0.001, OR = 1.730, 95% CI = 1.299–2.303). However, the other three* VDR* gene polymorphisms (*Bsm* I,* Taq* I, and* Apa *I) showed no statistically significant differences in the caries groups compared with the controls.

**Conclusion:**

* VDR-Fok *I gene polymorphisms may be associated with susceptibility to permanent tooth caries in Chinese adolescent.

## 1. Introduction

Dental caries is a polyfactorial infectious disease involving interplay between environmental factors and multiple genetic factors. Despite more than 100 years of continued prevention of this disease, caries is still a major oral problem throughout the world, affecting 60%–90% of schoolchildren [1]. As the economy is booming and sugar consumption is increasing in China, caries is nowadays the most popular oral disease in China [2].

The microbiological and environmental factors causing dental caries have been extensively studied. However, cariogenic microbial and environmental exposures are not sufficient to explain susceptibility to caries. Host susceptibility is highlighted by potential genetic factors for caries risk. More than 20 candidate genes have been reported, including enamel formation genes, immune response genes, and genes related to saliva, taste, and others [3].

Enamel is a hard and highly mineralized substance covering tooth crown and protects the tooth as a barrier. There were potential associations between genes responsible for enamel formation and susceptibility to caries. Previous studies suggest that the variation in genes encoding enamelin, tuftelin, and amelogenin may be related to susceptibility to dental caries [4, 5]. Recently, gene-linkage analysis suggests that several enamel-forming related genes may contribute to susceptibility to caries in different ethnic populations including Korean [6], Brazilian [7], and Japanese [8]. In contrast, Gasse et al. found that SNPs of in amelogenin gene are not associated with susceptibility to caries [9]. No association between mannose-binding lectin (MBL) and dental caries was proven in Chinese children [10] nor between amelogenin X (AMELX) gene and caries susceptibility in Polish children [11].

Vitamin D (Vit D) plays a significant rule in enamel mineralization [12] and the level of Vit D in serum is proved to be associated with caries [13]. Vitamin D receptor* (VDR)* is regarded as a mediator for the effect of Vit D related biomineralization.* VDR* is involved in biomineralization during mineralized tissue development, such as bone and tooth enamel [14, 15]. It has been indicated that the* VDR* gene has multiple polymorphisms, including the following four single nucleotide polymorphisms (SNPs):* Bsm *I (rs1544410),* Taq *I (rs731236),* Apa *I (rs7975232), and* Fok *I (rs10735810) [16]. Studies have shown that* VDR* gene polymorphisms may affect susceptibility to various diseases, such as osteoarthritis, diabetes, cardiovascular disease, and tuberculosis [17], as well as susceptibility to oral diseases, such as periodontitis [18] and dental implant loss [19].

Concerning the incidence of dental caries, gene immune deficiency and inflammation alterations may contribute to host susceptibility and affect the course of caries [20], so we speculated that* VDR* gene polymorphisms might have relationship with dental caries. In the present study, four SNPs of* VDR*,* Bsm *I (rs1544410),* Taq *I (rs731236),* Apa *I (rs7975232), and* Fok *I (rs10735810), were genotyped with the restriction fragment length polymorphism (RFLP) analysis method, aiming to find association between dental caries and* VDR *gene polymorphisms in Chinese adolescents.

## 2. Materials and Methods

### 2.1. Study Subjects

Our research was approved by the Ethics Committee of Guangzhou Medical University. It was performed under the guidelines of the World Medical Association Declaration of Helsinki. Informed consents were acquired from all adolescents and their guardians.

Four hundred Chinese adolescents aged 12 were recruited from the same city district primary schools for this study. Twelve-year-old adolescents were chosen for their permanent teeth, including second molars which had already erupted. Only permanent teeth were considered when the deciduous teeth still exist. Subjects were categorized into two groups according to the Decayed Missing Filled Teeth (DMFT) Index: caries-free (DMFT index = 0) and caries experience (DMFT index > 0). Each group contained two hundred members. Information about oral hygiene levels and habits including bleeding on probing (BOP), Volpe Manhold Index scores (VM-value), and brushing habit of each individual were collected at the same time.

### 2.2. Sample Collection

Two dentists conducted the clinical examinations. They used a periodontal probe and dental mirror according to the criteria recommended by the WHO guidelines. The diagnostic criterion of caries lesions was determined by visual examination of tooth surfaces. Values of Cohen's kappa for agreement were 0.90 between two dentists.

Buccal epithelial cells were obtained by rubbing the buccal mucosa with a swab. In accordance with the instructions of manufacturer, genomic DNA was extracted with the use of a commercial kit (Tiangen, Beijing, China) following the instructions of the manufacturer. Extracted DNA was aliquoted for each sample and stored at −20°C until PCR amplification.

### 2.3. SNP Selection and Genotyping

The primers to amplify the* VDR* gene were designed using Primer Premier 5 (Premier Biosoft Inter, Palo Alto, USA) and synthesized by Shanghai Chaoshi Co. (Shanghai, China). Four SNPs of* VDR* gene were analyzed:* Apa *I (rs7975232, located at intron 8, in the 3′ UTR),* Bsm *I (rs1544410, located at intron 8, in the 3′ UTR),* Taq *I (rs731236, located at intron 9, in the 3′ UTR), and* Fok *I (rs10735810, located at intron 2, at the first start codon). Genotyping was performed by the restriction fragment length polymorphism (RFLP) technique [21].  Table 1 presents the primers and their amplifying fragment lengths.

### 2.4. Statistical Analysis

Data for this study were analyzed with Statistical Package for the Social Sciences (SPSS) 18.0 statistical software (USA). A Hardy–Weinberg equilibrium assessment was performed. The difference in proportion of gender, age, and oral hygiene status between the caries group and the caries-free controls was compared by Chi-square test. The significance of allele, genotype frequency, and allele carriage rate differences between two groups were also evaluated by Chi-square test. The results were analyzed with 95% confidence interval (CI). Haploview4.2 (http://www.broadinstitute.org/haploview) was used to carry out linkage disequilibrium analysis (LD) and haplotype analysis. The values of LD were calculated with *D*′ and displayed with confidence bounds. The significance of different haplotypes was offered by permutation tests. The* p* value < 0.05 was selected to define statistical significance.

## 3. Results

The distribution of gender and age in caries group and caries-free group was equipoised. The female to male ratio was 1 : 0.89 and 1 : 1.04 for healthy controls and dental caries adolescents, respectively. The mean age of caries-free group and dental caries adolescents was 12.24 ± 0.30 years and 12.20 ± 0.40 years, respectively. The gender and mean age between the two subjects was not significantly different (*p* value > 0.05). The data of BOP, VM-value, brushing time, and frequency of the two analyzed groups showed no significant difference (*p* value > 0.05).  Table 2 illustrates the demographic characteristics of the analyzed groups.

Table 3 shows the distribution of genotypes and allele frequencies for* Bsm *I (rs1544410),* Taq *I (rs731236),* Apa *I (rs7975232), and* Fok *I (rs10735810) SNPs of* VDR* gene in the two analyzed groups. The results were fitted to a Hardy–Weinberg equilibrium (*p* value > 0.49).

Significant differences were detected in the frequencies of* Fok *I genotype between the caries group and the caries-free group. In particular, the frequency of* Fok *I's TT genotype was obviously lower in the caries experience group than that in the controls (9% versus 24.5%, resp.). We also found a lower carriage rate of allele T (57% in the caries group versus 67.5% in the controls, *p* value = 0.03) and a higher carriage rate of allele C (91% versus 75.5%, *p* value ≤ 0.001) in the caries group, compared to the caries-free controls. The odds ratio of the carriage of allele C reached 3.281, which showed a strong relationship between allele C of* Fok *I and caries group (OR > 3). We found that the frequency of allele C was markedly higher, while the frequency of allele T was significantly lower in the caries cases compared to the caries-free controls.

By contrast, for the other three* VDR* gene SNPS (*Bsm *I,* Taq* I, and* Apa *I), the genotypes and allele frequencies (G/A, T/C, and C/A, resp.) showed no statistically significant differences between the caries group and the control group.

**Figure 1:**
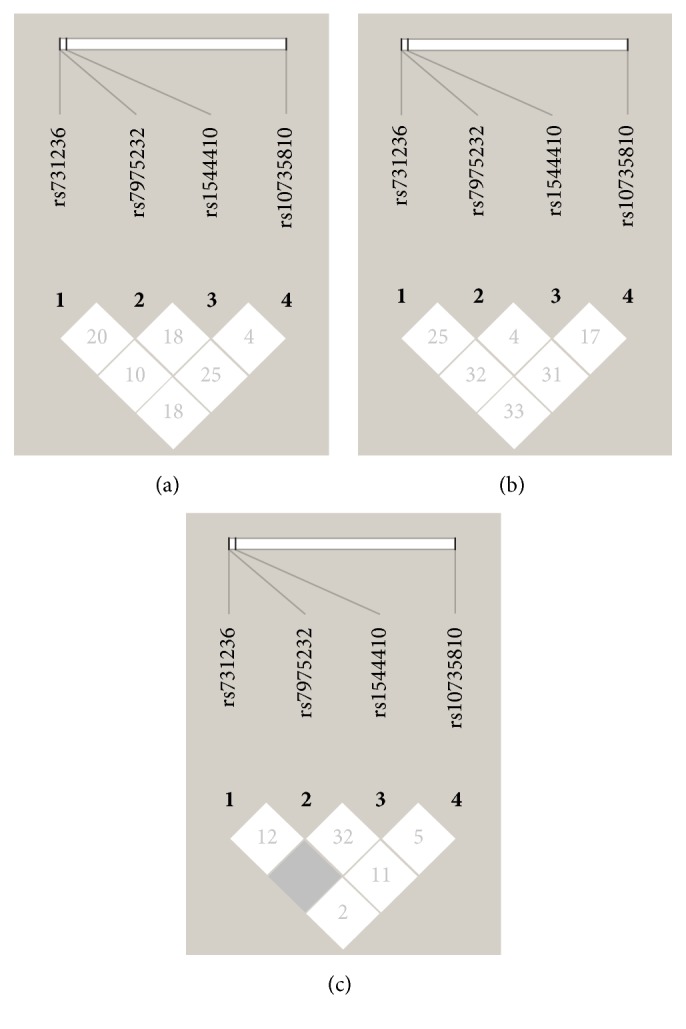
LD analysis of SNPs in caries and caries-free group. LD plot of SNPs* Taq *I (rs731236),* Apa *I (rs7975232),* Bsm *I (rs1544410), and* Fok *I (rs10735810) in caries and caries-free group (a), caries-free group only (b), and caries group only (c). All these four SNPs showed strong recombination of each other (block in white) in both caries and caries-free group (a) and caries-free group only (b), while linkage of* Taq *I (rs731236) and* Bsm *I (rs1544410) reached a higher level (block in gray, *D*′ = 1) in caries group only.

All these four SNPs showed strong evidence of recombination when we performed LD analysis on data of caries and caries-free group or caries-free group only. The linkage between* Taq *I and* Bsm* I reached an uninformative status, an intermediate state from strong recombination to strong linkage disequilibrium, when LD analysis was carried out on data of caries group only (Figure 1).

Haplotypes of these four SNPs were presented in an order based on the physical location of these four SNPs. The haplotypes TCGT and TAAC showed a significant difference between the cases and controls since their *p* values were far less than 0.05. But when we put these data into 999-time permutation tests, only haplotype TCGT and SNP* Fok* I still had significant difference between the cases and controls (Table 4).

## 4. Discussion 

Dental caries is a polyfactorial infectious disease involving interplay between environmental factors and multiple genetic factors. To rule out the effects of environmental factors in these two groups we compared, we collected information about oral hygiene levels and oral habits of each individual in cases and controls. There were no significant differences between case group and control group among these several factors (Table 2). This result offered support to the hypothesis that the differences of caries experience between cases and controls were possibly associated with genetic factors.

*VDR* gene polymorphisms are important factors for normal enamel development [12] and have been shown to contribute to susceptibility to various immune diseases [22]. Variations in human* VDR* gene lead to phenotypically diverse inherited enamel malformations [15]. Considering the role of the* VDR* gene in enamel formation, we hypothesized a possible contribution between* VDR* gene polymorphisms and dental caries. Our results support the hypothesis that susceptibility of Chinese adolescent to caries of permanent teeth was associated with the genotype frequency of the* Fok *I SNP.

Four SNPs of* VDR*,* Bsm *I (rs1544410),* Taq *I (rs731236),* Apa *I (rs7975232), and* Fok *I (rs10735810) were genotyped by the restriction fragment length polymorphisms analysis method in our study. Previous studies suggested that the distribution of* VDR* polymorphisms could have different patterns in different ethnicity [21, 23–26]. In this study, we failed to find any incidence of AA genotype for* Bsm *I and TT genotype for* Taq *I in Chinese adolescents. The most common genotype for each of the polymorphisms was GG for* Bsm *I (83.25%), CC for* Taq *I (82.25%), CC for* Apa *I (44.75%), and CT for* Fok *I (45.5%). The general distribution of* VDR *gene polymorphisms in our study showed similar pattern with previous study of Chinese population [21] but was different to those on African Americans [24], Turks [25], and Jordanians [26]. These consistency and inconsistency of our results with previous studies presented extra evidences to the existence of distribution specificity of* VDR *gene polymorphisms based on ethnicity and also suggested that the population we collected in this study could represent characteristics of Chinese to some extent.

Our findings showed that the susceptibility of Chinese adolescents to permanent tooth caries was associated with the genotype frequency of* Fok *I. The frequency of allele C was obviously higher in cases compared with the caries-free controls. The odds ratio of carriage of allele C reached 3.281, suggesting that allele C seems to be a risk factor of caries experience. The increased frequency of genotypes CT and CC were also found in caries group, while the genotype TT and allele T frequency were found to be significantly reduced in the caries group compared with the caries-free controls. These findings indicated that the allele T of* Fok *I appeared to be a protective factor for the caries experience, as the odd ratio and 95%CI of carriage rate of allele T was 0.638 (0.425–0.959) <1. In contrast, for the other three* VDR* genes (*Apa *I,* Bsm *I, and* Taq *I), the genotypes and allele frequencies (C/A, G/A, and T/C, resp.) showed no statistically remarkable differences between the caries group and the control group.

It is notable that the minor allele frequency (MAF) of* Bsm *I and* Taq *I was extremely low in CHB base on HapMap project since MAF of* Bsm *I was 0.022 and* Taq *I was 0.011 (http://hapmap.ncbi.nlm.nih.gov/). This could explain why we failed to find any incidence of AA genotype for* Bsm *I and TT genotype for* Taq *I. These results also suggested that the number of samples needed to finally confirm the association between* Bsm *I,* Taq *I and caries susceptibility could be hundred times of the sample amount in our study. HapMap project data showed that* Apa *I had a much higher MAF (0.321), but the power on* Apa *I in our data merely reached 43.1%, which means that our data on* Apa *I had greater possibility of being a false negative result, while power on* Fok *I reached a 97.1% strong level (Table 3).* Apa *I,* Bsm *I, and* Taq *I could have potential association with caries susceptibility, but our data cannot offer a positive support to this hypothesis.

A recent study of dental caries in northwestern Chinese population indicated that* Taq *I could act as a caries risk factor in middle-age adults [27], which seemed to be a challenge to our data. But firstly, the power on* Taq *I in our data was not strong enough to reject false negative. On the other hand, China is a multinational country with 55 ethnic minorities and the majority Han people, and many of the ethnic minorities are living in relatively remote areas of China such as the southwest and northwest of China. So, the detailed composition of northwestern Chinese population could be much more complex than ours. This might partially explain the inconsistency.

In LD analysis, all these four SNPs showed very strong evidence of recombination except for* Taq *I and* Bsm *I in caries group data. However, the linkage of* Taq *I and* Bsm *I in caries group still did not reach the level of strong LD. Haplotype analysis and further permutation tests revealed that haplotypes TCGT and* Fok *I each were more likely to have association with exposure factor, while the haplotype TCGT seemed to be a protective one for its frequency was higher in caries-free group.

It has not been clear about the mechanism of* VDR* polymorphisms acting to caries susceptibility.* VDR* takes part in regulating the expression of genes related to immune response, calcium homeostasis, and cellular differentiation and proliferation [28]. In* VDR* gene, the SNPs* Apa *I,* Bsm *I, and* Taq *I are situated at the corresponding 3′UTR genomic DNA region, indicating that it was impossible for these three SNPs to result in the variation of the protein structure of* VDR *[29]. This might be the mechanical reason that SNPs* Apa *I,* Bsm *I, and* Taq *I reveal no significant relationship with the risk of dental caries in our study. On the other hand, SNP* Fok* I is lying in the exon 4 of* VDR* gene and locating in the start codon of* VDR* isoforms 1 and 2. It is made up of a T-to-C change with the occurrence of an initiation codon (ATG). An alternative start site would be utilized once the C variant exists; it might produce* VDR *protein of different sizes [28, 30]. This was consistent with our haplotype analysis result as the protective haplotype TCGT had T of* Fok *I not C. However, 3′UTR was also related to mRNA including the mRNA localization and stability and also the translation efficiency [31].

This study is the first to analyze the association between* VDR* gene polymorphisms and the susceptibility of Chinese school adolescents to caries in permanent teeth. Our findings suggest that* Fok *I could be a potential risk factor for caries in this specific population group. This finding may contribute to our knowledge of dental caries in terms of etiology and treatment course.

## Figures and Tables

**Table 1:** The primers of vitamin D receptor *(VDR)* and band site of genotype.

SNP	Alleles	Primers	Fragment size (bp)	Digested fragment length (bp)
*Bsm *I (rs1544410)	G/A	5′-ATACCTACTTTGCTGGTTTGC-3′ 5′-AGCCCATCTCCATTCCTTG-3′	512	AA: 512 AG: 512, 315, 197 GG: 315, 197
*Taq *I (rs731236)	T/C	5′-AGCAGAGCAGAGTTCCAAGCAGA-3′ 5′-ATCTTGGCATAGAGCAGGTGGCT-3′	345	CC: 345 CT: 345, 260, 85 TT: 260, 85
*Apa *I (rs7975232)	C/A		740	AA: 740 AC: 740, 535, 205 CC: 535, 205
*Fok *I (rs10735810)	C/T	5′-AGCTGGCCCTGGCACTGACTCTGGCT-3′ 5′-ATGGAAACACCTTGCTTCTTCTCCCTC-3′	265	CC: 265 CT: 265, 196, 69 TT: 196, 69

**Table 2:** Demographic characteristics of the study subjects.

	Cases	Controls	*X* ^2^	*p* value
	(*n* = 200)	(*n* = 200)		
Mean age	12.20 ± 0.40	12.24 ± 0.30		
Gender (%)			0.640	0.424
Female	98 (49)	106 (53)		
Male	102 (51)	94 (47)		
BOP (+)	28 (14.0)	23 (11.5)	0.562	0.454
VM-value (+)	41 (20.5)	36 (18.0)	0.402	0.526
Brushing time				
<3 min	171 (85.5)	165 (82.5)	0.700	0.413
≥3 min	29 (14.5)	35 (17.5)		
Brushing frequency				
<2 times a day	95 (47.5)	91 (45.5)	0.161	0.688
≥2 times a day	105 (52.5)	109 (54.5)		

*Note*. BOP: bleeding on probing; VM-value: Volpe Manhold Index scores.

**Table 3:** Summary of allele and genotype frequencies in 12-year-old adolescents.

VDR gene polymorphisms (rs number)		Total	Caries experience (*n* = 200)	Caries-free (*n* = 200)	*X* ^2^	*p* value	OR (95% CI)	Power (%)
*Bsm *I (rs1544410)	AA	0	0	0	0.448	0.503		
	AG	67 (16.75)	36 (18)	31 (15.5)				
	GG	333 (83.25)	164 (82)	169 (84.5)				
	Carriage of allele A		36 (18)	31 (15.5)	0.448	0.503	1.197 (0.707–2.026)	
	A	67 (8.375)	36 (9)	31 (7.75)	0.407	0.523	1.177 (0.713–1.944)	9.8
	G	733 (91.625)	364 (91)	369 (92.25)				

*Taq *I (rs731236)	CC	329 (82.25)	171 (85.5)	158 (79)	2.894	0.089		
	CT	71 (17.75)	29 (14.5)	42 (21)				
	TT	0	0	0				
	Carriage of allele T		29 (14.5)	42 (21)	2.894	0.089	0.638 (0.379–1.073)	
	C	729 (91.125)	371 (92.75)	358 (89.5)	2.612	0.106	1.501 (0.915–2.462)	47.5
	T	71 (8.875)	29 (7.25)	42 (10.5)				

*Apa *I (rs7975232)	AA	57 (14.25)	33 (16.5)	24 (12)	2.898	0.235		
	AC	164 (41)	85 (42.5)	79 (39.5)				
	CC	179 (44.75)	82 (41.0)	97 (48.5)				
	Carriage of allele A		118 (59)	103 (51.5)	2.275	0.131	1.355 (0.913–2.012)	
	Carriage of allele C		167 (83.5)	176 (88)	1.657	0.198	0.69 (0.391–1.216)	
	A	278 (34.75)	151 (37.75)	127 (31.75)	3.175	0.075	1.304 (0.974–1.745)	43.1
	C	522 (65.25)	249 (62.25)	273 (68.25)				

*Fok *I (rs10735810)	CC	151 (37.75)	86 (43.0)	65 (32.5)	17.813	0.000		
	CT	182 (45.5)	96 (48.0)	86 (43.0)				
	TT	67 (16.75)	18 (9)	49 (24.5)				
	Carriage of allele C		182 (91)	151 (75.5)	17.229	0.000	3.281 (1.834–5.870)	
	Carriage of allele T		114 (57)	135 (67.5)	4.692	0.03	0.638 (0.425–0.959)	
	C	484 (60.5)	268 (67)	216 (54)	14.144	0.000	1.730 (1.299–2.303)	97.1
	T	316 (39.5)	132 (33)	184 (46)				

**(a):** 

Haplotype	Freq.	Case, control ratio counts	Case, control frequencies	*X* ^2^	*p* value
TCGC	0.309	132.7 : 267.3, 114.7 : 285.3	0.332, 0.287	1.901	0.168
TCGT	0.25	80.3 : 319.7, 119.5 : 280.5	0.201, 0.299	10.259	0.0014
TAGC	0.197	89.7 : 310.3, 67.9 : 332.1	0.224, 0.170	3.758	0.0525
TAGT	0.088	35.0 : 365.0, 35.4 : 364.6	0.087, 0.088	0.003	0.9596
TCAT	0.05	21.9 : 378.1, 18.4 : 381.6	0.055, 0.046	0.312	0.5764
CAGT	0.03	12.1 : 387.9, 12.1 : 387.9	0.030, 0.030	0	0.9992
CCGC	0.018	7.6 : 392.4, 6.5 : 393.5	0.019, 0.016	0.088	0.7669
CCGT	0.017	4.1 : 395.9, 9.2 : 390.8	0.010, 0.023	1.979	0.1595
TAAC	0.013	9.4 : 390.6, 1.2 : 398.8	0.023, 0.003	6.391	0.0115

**Table:** (b) #999 permutations performed

Name	*X* ^2^	Permutation *p* value
TCGT	10.259	0.016
*Fok *I (rs10735810)	8.544	0.026
TAAC	6.391	0.0581
TAGC	3.758	0.3033
*Apa *I (rs7975232)	3.175	0.4765
*Taq *I (rs731236)	2.612	0.6056
CCGT	1.979	0.7217
TCGC	1.901	0.7598
*Bsm *I (rs1544410)	0.407	0.998
TCAT	0.312	0.998
TAGT	0.003	1
CAGT	0	1
CCGC	0.088	1

## References

[1] Petersen P. E. (2003). The World Oral Health Report 2003: continuous improvement of oral health in the 21st century—the approach of the WHO Global Oral Health Programme. *Community Dentistry and Oral Epidemiology*.

[2] Wang J.-D., Chen X., Frencken J., Du M.-Q., Chen Z. (2012). Dental caries and first permanent molar pit and fissure morphology in 7- to 8-year-old children in Wuhan, China. *International Journal of Oral Science*.

[3] Vieira A. R., Modesto A., Marazita M. L. (2014). Caries: Review of human genetics research. *Caries Research*.

[4] Patir A., Seymen F., Yildirim M. (2008). Enamel formation genes are associated with high caries experience in Turkish children. *Caries Research*.

[5] Deeley K., Letra A., Rose E. K. (2008). Possible association of amelogenin to high caries experience in a Guatemalan-Mayan population. *Caries Research*.

[6] Kang S., Yoon I., Lee H., Cho J. (2011). Association between AMELX polymorphisms and dental caries in Koreans. *Oral Diseases*.

[7] Jeremias F., Koruyucu M., Küchler E. C. (2013). Genes expressed in dental enamel development are associated with molar-incisor hypomineralization. *Archives of Oral Biolog*.

[8] Ohta M., Nishimura H., Asada Y. (2014). Association of DLX3 gene polymorphism and dental caries susceptibility in Japanese children. *Archives of Oral Biolog*.

[9] Gasse B., Grabar S., Lafont A. G. (2013). Common SNPs of amelogeninX (AMELX) and dental caries susceptibility. *Journal of Dental Research*.

[10] Yang Y., Wang W., Qin M. (2013). Mannose-binding lectin gene polymorphisms are not associated with susceptibility to severe early childhood caries. *Human Immunology*.

[11] Olszowski T., Adler G., Janiszewska-Olszowska J., Safranow K., Kaczmarczyk M. (2012). MBL2, MASP2, AMELX, and ENAM gene polymorphisms and dental caries in Polish children. *Oral Diseases*.

[12] Berdal A., Lézot F., Néfussi J. R., Sautier J. M. (2000). Mineralized dental tissues: a unique example of skeletal biodiversity derived from cephaic neural crest. *Morphologie : bulletin de l"Association des anatomistes*.

[13] Schroth R. J., Rabbani R., Loewen G., Moffatt M. E. (2016). Vitamin D and Dental Caries in Children. *Journal of Dental Research*.

[14] Mesbah M., Nemere I., Papagerakis P. (2002). Expression of a 1,25-dihydroxyvitamin D3 membrane-associated rapid-response steroid binding protein during human tooth and bone development and biomineralization. *Journal of Bone and Mineral Research*.

[15] Zhang X., Rahemtulla F., Zhang P., Beck P., Thomas H. F. (2009). Different enamel and dentin mineralization observed in VDR deficient mouse model. *Archives of Oral Biolog*.

[16] Ben-Selma W., Ben-Fredj N., Chebel S., Frih-Ayed M., Aouni M., Boukadida J. (2015). Age- and gender-specific effects on VDR gene polymorphisms and risk of the development of multiple sclerosis in Tunisians: A preliminary study. *International Journal of Immunogenetics*.

[17] Valdivielso J. M., Fernandez E. (2006). Vitamin D receptor polymorphisms and diseases. *Clinica Chimica Acta*.

[18] Martelli F. S., Martelli M., Rosati C., Fanti E. (2014). Vitamin D: Relevance in dental practice. *Clinical Cases in Mineral and Bone Metabolism*.

[19] Alvim-Pereira F., Montes C. C., Thomé G., Olandoski M., Trevilatto P. C. (2008). Analysis of association of clinical aspects and vitamin D receptor gene polymorphism with dental implant loss. *Clinical Oral Implants Research*.

[20] Rashid M., Zarkadas M., Anca A., Limeback H. (2011). Oral manifestations of celiac disease: A clinical guide for dentists. *The Journal of the Michigan Dental Association*.

[21] Pei F. H., Wang Y. J., Gao S. L. (2011). Vitamin D receptor gene polymorphism and ulcerative colitis susceptibility in Han Chinese. *Journal of Digestive Diseases*.

[22] Uitterlinden A. G., Fang Y., Van Meurs J. B. J., Van Leeuwen H., Pols H. A. P. (2004). Vitamin D receptor gene polymorphisms in relation to Vitamin D related disease states. *The Journal of Steroid Biochemistry and Molecular Biology*.

[23] O'Neill V., Asani F. F., Jeffery T. J., Saccone D. S., Bornman L. (2013). Vitamin D Receptor Gene Expression and Function in a South African Population: Ethnicity, Vitamin D and FokI. *PLoS ONE*.

[24] Sarkissyan M., Wu Y., Chen Z. (2014). Vitamin D receptor FokI gene polymorphisms may be associated with colorectal cancer among African American and Hispanic participants. *Cancer*.

[25] Karatayli S. C., Ulger Z. E., Ergul A. A. (2014). Tumour necrosis factor-alpha, interleukin-10, interferon-gamma and vitamin D receptor gene polymorphisms in patients with chronic hepatitis delta. *Journal of Viral Hepatitis*.

[26] Karasneh J. A., Ababneh K. T., Taha A. H. (2013). Association of vitamin D receptor gene polymorphisms with chronic and aggressive periodontitis in Jordanian patients. *European Journal of Oral Sciences*.

[27] Hu X. P., Li Z. Q., Zhou J. Y., Yu Z. H., Zhang J. M., Guo M. L. (2015). Analysis of the association between polymorphisms in the vitamin D receptor (VDR) gene and dental caries in a Chinese population. *Genetics and Molecular Research*.

[28] Uitterlinden A. G., Fang Y., van Meurs J. B. J., Pols H. A. P., van Leeuwen J. P. (2004). Genetics and biology of vitamin D receptor polymorphisms. *Gene*.

[29] Li S., Yang M. H., Zeng C. A. (2008). Association of vitamin D receptor gene polymorphisms in Chinese patients with generalized aggressive periodontitis. *Journal of Periodontal Research*.

[30] Tanaka K., Miyake Y., Hanioka T., Arakawa M. (2013). VDR gene polymorphisms, interaction with smoking and risk of periodontal disease in Japanese women: The Kyushu Okinawa maternal and child health study. *Scandinavian Journal of Immunology*.

[31] Mayr C. (2016). Evolution and Biological Roles of Alternative 3'UTRs. *Trends in Cell Biology*.

